# The course of pain drawings during a 10-week treatment period in patients with acute and sub-acute low back pain

**DOI:** 10.1186/1471-2474-7-65

**Published:** 2006-08-11

**Authors:** Marie Grunnesjö, Johan Bogefeldt, Stefan Blomberg, Heléne Delaney, Kurt Svärdsudd

**Affiliations:** 1 Uppsala University, Department of Public Health and Caring Sciences, Family Medicine and Clinical Epidemiology Section, SE-751 85 Uppsala, Sweden; 2 Stockholm Clinic – Stay Active, Stockholm, Sweden

## Abstract

**Background:**

Pain drawings are widely used as an assessment of patients' subjective pain in low back pain patients being considered for surgery. Less work has been done on primary health care patients. Moreover, the possible correlation between pain drawing modalities and other pain assessment methods, such as pain score and functional variables needs to be described. Thus, the objectives were to describe the course of pain drawings during treatment in primary health care for low back pain patients.

**Methods:**

160 primary health care outpatients with acute or sub-acute low back pain were studied during 10 weeks of a stay active concept versus manual therapy in addition to the stay active concept. The patients filled out 3 pain drawings each, at baseline and after 5 and 10 weeks of treatment. In addition the patients also reported pain and functional variables during the 3 measurement periods.

**Results:**

The proportion of areas marked, the mean number of areas marked (pain drawing score), mean number of modalities used (area score), and the proportion of patients with pain radiation all decreased during the 10-week treatment period. Most of the improvement occurred during the first half of the period. The seven different pain modalities in the pain drawing were correlated to pain and functional variables. In case of no radiation some modalities were associated with more pain and disability than others, a finding that grew stronger over time. For patients with pain radiation, the modality differences were smaller and inconsistent.

**Conclusion:**

Pain modalities are significantly correlated with pain and functional variables. There is a shift from painful modalities to less painful ones over time.

## Background

Low-back pain is a major diagnostic and therapeutic problem, which causes a great deal of suffering and is a major expense to society [1,2]. Ever since 1949 when Harold Palmer [3] suggested pain drawings to distinguish functional pain from organic pain, the pain drawing has appealed to the medical society due to its possibility to describe relatively complex pain experiences and yet be simple for the patient to use. The pain drawing sketch is a body silhouette filled out with various symbols representing different types of pain modalities such as 'stabbing pain' and 'cramps'. The pain drawings have been subjected to more than 30 years of interest as a screening tool, and have been used in various contexts, with various objectives, for instance as a single assessment method for measuring the proportion of the body surface affected by pain [4-6]. Pain drawings have been shown to be reliable instruments over time for evaluation of the course of pain in chronic pain patients [4].

Pain drawings have also been shown to be associated with both pain and functional variables or pain related disability [7-9]. For example, both the Oswestry total score and the Roland-Morris disability index correlate with the quantified pain drawing [8,9]. However, this does not apply to qualitative aspects of the pain drawing, such as individual pain modalities. No association between the various pain modalities and pain or functional variables has been shown in patients with acute or sub-acute low back pain. If such an association exists it would add further information to the pain drawing, especially regarding the interpretation of the course of the low back pain. Moreover, since it is well known that severe radiating leg pain affects the course of the low back pain negatively [10] radiation might confound a possible association between pain and function on the one hand and pain modalities on the other.

We performed a randomized controlled trial in primary health care outpatients, where the alternative treatments were a stay active concept versus manual treatment in addition to the stay active concept. Some of the main results have been reported previously [11]. The objectives of this report were firstly to investigate how pain drawing markings change over time among non-selected primary health care patients with sub-acute low back pain regardless of type of treatment, secondly to correlate the various pain modalities with self-reported pain and functional variables and thirdly to study the influence of pain radiation on the pain-modality-function.

## Methods

### Design and sampling

The sampling procedure and the methods used have been described in detail elsewhere [11,12]. Briefly, the study was performed in the province of Gotland, Sweden, from January 1994 to December 1998 with recruitment during 32 months of the period. Only patients with symptoms severe enough to motivate seeing a doctor were objects of the recruitment. The recruitment general population segment (n = 19,000) consisted of persons 20–55 years of age, employed and with no threat of job loss, born in Sweden and sufficiently articulate not to jeopardize the verbal contact with physicians or physiotherapists. The additional inclusion criteria were:

- Acute or sub-acute low back pain (i.e. back pain below the level of the seventh thoracic vertebra) with or without pain radiating to one or both legs, not requiring surgical or rheumatologic care. Patients with demonstrated or suspected herniated disc were included if surgery was not being considered. Low back pain was to dominate the clinical condition but other musculoskeletal symptoms, not requiring sick leave, were allowed.

- Symptom duration of 3 months or less, preceded by at least 2 months of relative freedom from symptoms.

- Consent to treatment and follow up for ten weeks.

- Agreement not to consult therapists other than those participating in the study during the ten weeks.

- Absence of other conditions or circumstances that might jeopardize completion of treatment and follow up (e.g. pregnancy, malignant tumours, alcoholism or severe psychiatric disorders).

- No previous treatment of current complaints with specific mobilization or manipulation.

- No previous participation in the study.

All patients preliminarily fulfilling the inclusion criteria were referred by general practitioners (GPs) at primary health care centres or by physicians at Visby hospital to the recruiting physician in the study. In addition, in order to secure an unselected study population the National Social Insurance Office, a government agent that handles all sick leave compensation for periods two weeks or longer, was engaged to refer all patients that were filing sick leave applications for low back pain. The recruiting physician examined all patients and made the final assessment of whether or not they fulfilled the inclusion criteria.

Of the 316 patients referred, 111 did not fulfil the inclusion criteria, and 45 declined to participate. The remaining 160 patients were included after informed consent was obtained, and were allocated to the treatment groups, using sealed pre-prepared envelopes with group assignment derived from a random table.

### Treatment

The treatments given have been described in detail elsewhere [11]. Briefly, the stay active care concept [13] was the basic management strategy in the groups. The patients were informed of the benign nature of their condition and the adverse effects of inactivity and sick leave, and were encouraged to take part in physical and other activities. In addition to the stay active care concept, treatment was provided by physiotherapists and physicians individually, in groups or both. Treatment modalities were chosen from a group specific 'toolbox' after clinical assessment of the patients and according to need.

Two orthopaedic surgeons at Visby Hospital and eight physiotherapists treated the reference patients. In accordance with the study design, muscle stretching was a treatment option in 51% of the reference group, and 41% actually received muscle stretching. Two GPs based at primary health care centres in Visby and nine physiotherapists treated the experimental patients. They received the full reference treatment, plus specific mobilization, spinal manipulation, and auto-traction when indicated. According to the study design steroid injections were a treatment option in 52% of the experimental group, but less than half of these patients received injections.

### Data collection

Data on patient characteristics were recorded at baseline and data on pain, pain drawings, disability index and other variables measuring the course of the disease were measured using a questionnaire answered on location at baseline, and with postal questionnaires after 5 and 10 weeks of treatment.

### Pain drawings

**Figure 1:**
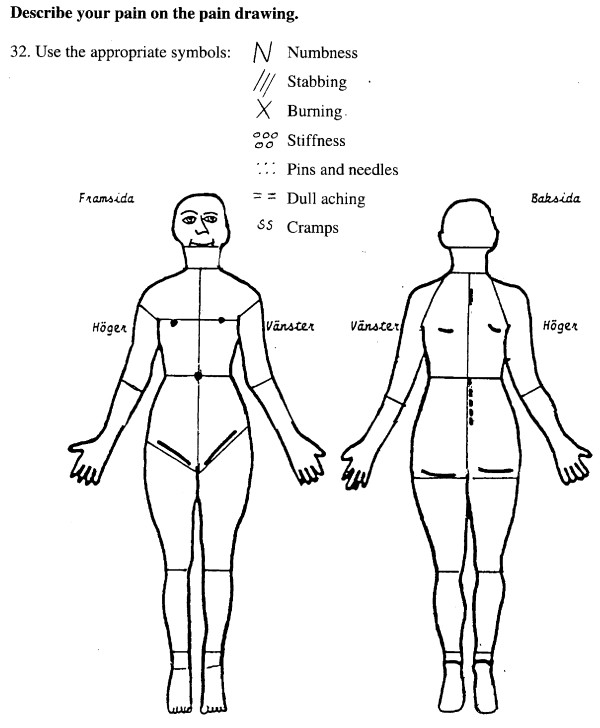
**Pain drawing sketch**. The pain drawing sketch with its 34 anatomical areas, and the seven pain modalities and their symbols.

The pain drawing sketch contained 34 anatomical areas, 8 dorsal and 8 frontal areas below the waistline and the same numbers above the waistline, Figure 1. The patients were instructed to describe their pain intensity and quality in each of these areas by markings with seven pain modality symbols. All marks were recorded by modality and ranked for dominance by the same observer. The pain modality most frequently used in each area was considered to be the dominating one. The dominating pain modality in the low back pain area was defined as the most frequently used pain modality in the left and right lower back/buttock areas together. Pain drawing score (PDS) [14] was assessed as the mean number of areas with at least one mark, range 0 to 34. Pain radiation was extracted from the pain drawings. The degree of pain radiation to the legs was classified as no radiation, i.e. pain confined to the lower back/buttock area, or radiation to the leg, which was defined as pain in the lower back/buttock and in at least one area in the leg, frontal or dorsal side. The number of pain modalities used in each area, the 'area score', was also assessed [15,16]. Finally, patients with no marks above waistline were assessed for the dominating pain modality in all 16 areas below waistline. This limitation was done to minimize the confounding effect from other painful areas since the pain score and the disability rating index both are global scores.

### Pain score and disability rating index

The average pain experience during the previous week was used as the pain score. The disability rating index [17] is the mean score of twelve daily activity functional variables (physical exercise or sports, running, heavy physical work, heavy lifting, carrying a bag, leaning over a wash-stand, making a bed, moderate physical work, walks, mounting stairs, sitting still more than briefly and dressing or undressing). Pain score and the disability rating index were measured with visual analogue scales, 100 millimetres long, ranging from 0 (no pain or problem) to 100 (maximum pain or problem). The distance in millimeters from the left end of the scale to the patient's marking was used as the pain or disability rating score. The Research Ethics Committee of the Faculty of Medicine at Uppsala University approved the study.

### Data analysis

Data were analyzed with the JMP [18] and SAS [19] statistical programme packages. Data loss owing to missing data was less than 1%. All analyses were done in the complete study population regardless of treatment group. Summary statistics, such as means, dispersions and confidence intervals were computed using standard parametric methods. The analyses of association were done with standard least square analyses and one-way analysis of variance with pain score or disability rating index, respectively, as dependent variables and PDS as the independent variable at 0, 5 and 10 weeks and for the whole period. In the latter analysis the data from the various time points were stacked. The results from the separate time points and the overall period were consistent. The regression surface in figure 3 was constructed using multivariate linear regression technique with pain score or disability rating index as dependent variable and the seven separate pain modalities and pain radiation as independent variables. The analysis was performed on the 436 pain drawings with marks only below waistline among the 480 possible across the study period to eliminate the possibility of influence on pain score and disability index from painful sites above the waistline. Only two-tailed tests were used. P-values less than 5% were considered to indicate statistical significance.

## Results

### Patient characteristics

The patients were on average 41 years old (range 20–55), 44% were females, and 44% were cigarette smokers. 69% were on sick leave at baseline. In addition, 74% had been on sick leave due to low back pain in the last two years, approximately 80% of these for one month or less.

### Pain distribution

At baseline, the proportion of patients with marked areas ranged from 85.6% in the left lower back/buttock area to 3.1% in the right dorsal foot area, Table 1. The left side generally had a higher proportion of marked areas than the right side. In all areas but the frontal side of the lower leg and foot there was a large drop in the proportion of patients with marked areas during the first five weeks, on average 34.5 per cent units, and then a more moderate decrease during the next five weeks, on average 6.0 per cent units. The pattern was similar in all treatment groups. At baseline the pain drawing score was 3.7 for all areas (range 1–14) and 3.6 for the 16 areas below waistline (range 0–12). During the first five weeks this score dropped by approximately 33% and then remained stable (range 0–10). At baseline 70.9% of the patients had pain radiating to the knee and 38.6% to the lower leg. At the 10-week follow up the corresponding frequencies were 24.1% and 22.2%.

The mean number of pain modalities, the area score, is shown in Table 2. At baseline the area score ranged from 1.79 in the lower left back/buttock to 0.03 in the dorsal side of the right foot. As for the previously presented measures, the mean number of modalities used decreased over the 10-week period, but most of the reduction, on average 46.2%, occurred during the first half of the period.

**Figure 2:**
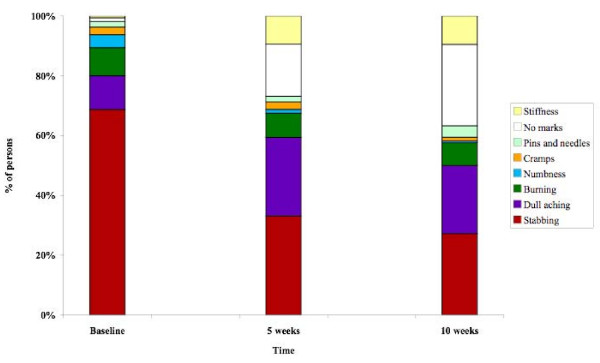
**Pain modality distribution**. Distribution of pain modalities in the lower back/buttocks area at baseline and after 5 and 10 weeks of follow up.

There was a shift of dominating pain modality prevalence in the lower back/buttock areas during the follow up, Figure 2. Stabbing pain decreased from 66.9% at baseline to 27.2% at 10 weeks follow up and 'no marks' increased from 1% to 27%.

### Pain drawings, pain and functional variables

There was an association between, on the one hand, mean number of areas marked, i.e. the pain drawing score, and pain score during the previous week (r = 0.39, p < 0.0001), and the disability rating index (r = 0.40, p < 0.0001) on the other, across the study period. Pain radiation was present in 149 (34.2%) of the 436 pain drawings with no marks above the waistline. Among the latter, 'stabbing pain' was the most frequently used dominating pain modality (45.6%) followed by 'dull aching' (30.2%), 'stiffness' (12.1%), 'burning' (6.0%), 'numbness' (2.7%), 'pins and needles' (2.0%) and finally 'cramps' (1.4%).

**Figure 3:**
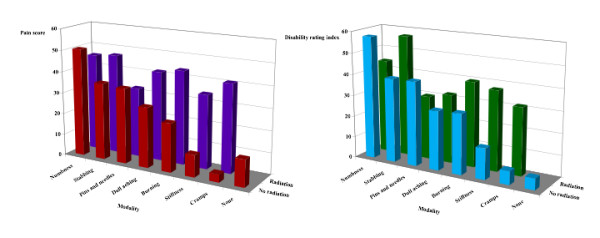
**Pain score and disability rating index**. Mean pain score and mean disability rating index in groups according to dominating pain modality and pain radiation, among patients with marks only below the waistline across the 10 week period.

Among the pain drawings with no pain radiation, there were no significant differences in pain score or disability rating index between the various pain modalities at baseline (p = 0.25 for the whole model). However, during the course of the study strong associations developed between pain modalities and pain score or disability rating (p < 0.0001 for the whole model at 5 and at 10 weeks). In Figure 3 the average pain score and disability rating index per modality across the 10-week period is displayed. The pain modality 'numbness' was associated with both the highest pain score and highest disability rating index, followed by 'pins and needles' and 'stabbing'. The pain modalities 'stiffness' and 'cramps' were associated with the least pain and least disability. For the pain drawings with pain radiation the differences were smaller and more inconsistent than in the non-radiation group.

## Discussion

The pain drawings were improved over time, an improvement that mainly occurred during the first half of the period. Also, in the pain drawings with no pain radiation some pain modalities were associated with more pain and disability than others, a finding that grew stronger over time. For the pain drawings with pain radiation, the differences were smaller and less consistent than for those with no radiation.

The study population was population based and the methods used are all well established and evaluated. All patient with low back pain seeking medical attention or receiving sick leave compensation in the area were assessed regarding inclusion in the study. The study population is therefore most certainly representative for this type of low back patients. There were no drop outs and the data loss owing to missing values or non-completed questionnaires or pain drawing sketches was minimal. We therefore have no reason to believe that the data would be biased to an extent affecting the conclusions.

To assess the pain drawing we used the pain drawing score, a frequently used method first described in 1986 by Margolis [14], and later used in numerous studies as an instrument to measure low back pain. Originally, the pain drawing score was based on anatomical regions within the body contour and on weights to compensate for the difference in area size. However, Margolis and others have suggested that the weights are unnecessary, since the raw scores correlate closely with the weighted scores (r = 0.97–0.99) [4,5].

Several studies on the validity and reproducibility of pain drawings have been published. It has been asserted that the instrument is sufficiently well-evaluated with regard to validity [8,20], that it is valid, reproducible and stable over time [4], and has low inter-rater variation [6,14]. Validity regarding pain, function and some psychological instruments is high [7,20]. Some authors have questioned the reliability of the pain drawing [4,6,20], while others have found the scoring system to have an acceptable reliability even with untrained observers [14].

Among pain drawing sketch variants available we chose Margolis', containing 43 areas all within the body contour. However, we moderated the anatomical areas size and number to a total of 34 areas for the present study population since the lower body has a higher impact and the area regions are more relevant for the purposes of our study. This system allows the pain to be scored using clinically reported pain patterns [21] and the system is easy to use. Since the patients in the present study were all suffering from low back pain, the 16 areas of the lower half of the body were of particular interest. Therefore, both the overall score and the 16 areas score below the waistline were reported.

The pain drawings were completed in the same way at all three measurement points. Two pain drawing patterns were prominent: the left side dominated the drawings, and there was a more substantial rate of improvement during the first period than the second. The left side domination has not previously been reported, and therefore remains to be replicated in other studies. The improvement manifested itself as fewer marked areas, fewer marks in the areas, fewer used modalities and more proximal marking, i.e., fewer marks in the leg areas. The early improvement is probably attributable to the fact that the majority of treatments were given in the first half of the 10-week treatment period [11]. The effects are seen in association with the treatment. The faster change in the first 5 weeks was also evident in the 'pain modality shift'.

The 'pain modality shift' as described by McKenzie [22], was seen in the low back/buttock area. The initially frequent modalities 'numbness' and 'stabbing' shifted to 'stiffness' and 'no marks' over time. There are some reports on the distribution of pain modalities in different regions [5,23]. However, they are based on a single measurement, making it impossible to deduce a 'pain modality shift' or the average speed at which the shift occurs.

In the present report there was a strong correlation (p < 0.0001) between self-rated pain, functional score and pain drawing score. These results are in line with the literature both for the disability rating [7-9] and the self-rated pain score [7,9,15]. To our knowledge there are no other studies that have analysed the possible association between various pain modalities and the grade of pain radiation on the pain drawing and pain score or disability rating. Furthermore, to compensate for the fact that pain score and disability rating index are global estimates, the association with the dominating leg pain modalities was analysed only in patients with all marks below waistline. The present study population had quite a homogenous pain drawing pattern, with the majority of marks below the waistline. Also, both the pain score and the disability rating index was low for patients with no marks in the lower back/buttock areas. We therefore have no reason to believe that the results are biased to an extent that could affect the conclusions.

Among patients who had no pain radiation there was a clear change towards fewer pain modalities and an emerging difference between modalities in pain score and disability index during the course of the study. This was not found among those who had pain radiation, a finding not described elsewhere to our knowledge. A possible explanation might be that since various pain modalities communicate with the brain through different type of fibres, patients who use a number of modalities to describe their pain might have difficulties in grading the sensations, and the addition of pain radiation makes the distinction between pain modalities too complex [2]. Thus, it is difficult to rate complex pain in a small area, and to rate radiating pain with different pain modalities in different areas is even more difficult, because other factors such as psychological coping strategies are likely to contribute to the rating. Also, there could be a minor change in choice of pain modality, a change that is too small to distinguish in this relatively small sample. However, if that is the case, one might ask whether such a minor change is clinically relevant.

The results from this study emphasize that the pain drawings should be part of the clinical practice when assessing sub-acute low back pain, as recommended in the biopsychosocial pain analyze for chronic pain [24]. Pain drawing information of the different pain modalities adds vital information, i.e. the patients describing pain modalities that are more painful should be subjected to a thorough biological investigation. The recognition that sub-acute low back pain patients with radiating pain are less likely to be able to rank the pain modalities needs further investigation, since radiating leg pain is described as a significant predictor to develop chronic back pain [10]. Also, by pain drawing patterns and choice of pain modality differentiate patient categories that benefits from early treatment or extended examination requires further investigation.

## Conclusion

The patients tended to use fewer pain modalities in their pain drawings over time and there was a shift from painful modalities to less painful ones. Most of the changes occurred during the first half of the study course. In the pain drawings of patients with no pain radiation the description of low back pain quality and extent was associated with both pain score and disability rating index. In patients with radiation the differences in pain score and disability index between modalities were smaller and inconsistent.

## Competing interests

The author(s) declare that they have no competing interests.

## Authors' contributions

SB designed the study and coordinated the data collection. SB, MG, JB and HD prepared the data before analysis. the statistical analysis and drafted the manuscript. participated in the data preparation. MG and KS performed the statistical analyses and drafted the manuscript. All authors participated in the manuscript revisions and read and approved the final version of the manuscript.

## Pre-publication history

The pre-publication history for this paper can be accessed here:



## Figures and Tables

**Table 1:** Pain drawing characteristics. Pain drawing characteristics at baseline, 5 and 10 weeks of follow up. 95% CI = 95% confidence intervals.

	Baseline		5 weeks follow up		10 weeks follow up	
			
	% or mean	95% CI	% or mean	95% CI	% or mean	95% CI
N	160		160		158	
*Proportion of areas marked, %*						
Dorsal side						
Lower back/buttock left	85.6	78.7–89.7	64.4	56.7–71.4	58.8	51.0–66.1
Lower back/buttock right	78.1	71.1–83.8	68.1	60.6–74.8	59.4	51.6–66.7
Thigh back, left side	38.1	31.0–45.8	16.2	11.3–22.7	15.6	10.8–22.0
Thigh back, right side	33.1	26.3–40.7	12.5	8.2–18.5	10.6	6.7–16.4
Lower leg back, left side	19.4	14.0–26.2	9.4	5.8–14.9	11.2	7.2–17.1
Lower leg back, right side	15.0	10.3–21.3	7.5	4.3–12.6	8.1	4.8–13.4
Plantar area left side	10.6	6.7–16.4	6.2	3.4–11.1	8.1	4.8–13.4
Plantar area right side	6.2	3.4–11.1	3.1	1.3–7.1	4.4	2.1–8.7
Frontal side						
Lower abdomen, left side	8.1	4.8–13.4	5.6	3.0–10.3	4.4	2.1–8.7
Lower abdomen, right side	8.1	4.8–13.4	3.7	1.7–7.9	3.7	1.7–7.9
Thigh front, left side	16.2	11.3–22.7	8.1	4.8–13.4	7.5	4.3–12.7
Thigh front, right side	15.6	10.8–22.0	6.2	3.4–11.1	5.6	3.0–10.3
Lower leg front, left side	6.9	3.9–11.9	6.2	3.4–11.1	6.9	3.9–11.9
Lower leg front, right side	4.4	2.1–8.7	3.1	1.3–7.1	4.4	2.1–8.7
Dorsal foot, left side	8.7	5.3–14.2	5.6	3.0–10.3	5.0	2.6–9.6
Dorsal foot, right side	3.1	1.3–7,1	2.5	1.0–6.3	1.9	0.6–5.4
All other areas	5.6	3.0–10.3	12.5	8.2–18.5	9.4	5.8–14.9
*Mean number of areas marked*						
Areas above waistline	0.1	0.03–0.17	0.3	0.15–0.45	0.26	0.11–0.42
Areas below waistline	3.6	3.3–3.8	2.3	2.0–2.6	2.2	1.8–2.5
All areas	3.7	3.4–4.0	2.6	2.3–2.9	2.4	2.0–2.8
*Radiation, any degree, %*						
Pain in lower back/buttock	100.0	97.6–100.0	83.5	77.0–88.5	72.8	65.4–79.1
Pain radiating to the thigh	70.9	63.4–77.4	32.9	26.1–40.6	24.1	18.1–31.3
Pain radiating to lower leg	38.6	31.4–46.4	22.2	16.4–29.2	22.2	16.4–29.2

**Table 2:** Modalities. Number of modalities, the 'area score', reported in the sixteen lower areas, group at baseline, 5 and 10 weeks of follow up. 95% CI = 95% confidence intervals

	Number of modalities used					
	
	Baseline		5 weeks follow up		10 weeks follow up	
			
	mean	95% CI	mean	95% CI	mean	95% CI
N	160		160		158	
Dorsal side						
Lower back/buttock left	1.79	1.63–1.95	1.02	0.87–1.17	0.91	0.76–1.06
Low. back/buttock right	1.61	1.43–1.78	1.01	0.88–1.15	0.92	0.78–1.07
Thigh back, left side	0.57	0.43–0.70	0.27	0.16–0.38	0.25	0.14–0.36
Thigh back, right side	0.48	0.36–0.61	0.17	0.09–0.24	0.14	0.07–0.21
Lower leg back, left side	0.27	0.18–0.37	0.14	0.06–0.21	0.17	0.08–0.27
Lower leg back, right side	0.22	0.14–0.31	0.09	0.04–0.14	0.11	0.05–0.17
Plantar area left side	0.14	0.07–0.20	0.10	0.04–0.16	0.10	0.04–0.16
Plantar area right side	0.08	0.03–0.13	0.05	0.01–0.10	0.06	0.01–0.10
Frontal side						
Lower abdomen, left side	0.11	0.05–0.18	0.08	0.02–0.14	0.07	0.01–0.13
Lower abdomen, right side	0.12	0.05–0.20	0.06	0.01–0.11	0.06	0.01–0.11
Thigh front, left side	0.24	0.15–0.34	0.12	0.05–0.19	0.14	0.05–0.24
Thigh front, right side	0.21	0.13–0.28	0.09	0.03–0.16	0.09	0.02–0.15
Lower leg front, left side	0.12	0.05–0.20	0.07	0.03–0.12	0.10	0.03–0.17
Lower leg front, right side	0.05	0.02–0.08	0.04	0.01–0.07	0.04	0.01–0.08
Dorsal foot, left side	0.13	0.05–0.21	0.07	0.02–0.13	0.07	0.02–0.13
Dorsal foot, right side	0.03	0.01–0.06	0.04	0.00–0.08	0.02	0.00–0.04
All other areas	0.13	0.03–0.23	0.38	0.17–0.58	0.39	0.13–0.65
